# Supplementary material for: Effect of Zwitterionic Additive on Electrode Protection through Electrochemical Performances of Anatase TiO2 Nanotube Array Electrode in Ionic Liquid Electrolyte
Source: Int J Mol Sci. 2023 Feb 9;24(4):3495. doi: 10.3390/ijms24043495 (PMC9966853; doi:10.3390/ijms24043495)
Supplement: Supplementary file 1 [file ijms-24-03495-s001.zip › ijms-2183421-supplementary.pdf]

Supporting Information

Effect of Zwitterionic Additive on Electrode Protection through Electrochemical Performances of Anatase TiO_2 Nanotube Array Electrode in Ionic Liquid Electrolyte

Aleksandra Roganović [1], Milan Vraneš [1], Nikola Cvjetićanin [2], Xiaoping Chen [3] and Snežana Papović [1,*]

[1] Faculty of Sciences, Department of Chemistry, Biochemistry and Environmental Protection, University of Novi Sad, Trg Dositeja Obradovića 3, 21000 Novi Sad, Serbia
[2] Faculty of Physical Chemistry, University of Belgrade, Studentski trg 12-16, 11158 Belgrade, Serbia
[3] Hangzhou Bay Automotive College/Mechanical College, Ningbo University of Technology, No.769, Bihai Road 2, 315336, Hangzhou Bay New Zone, 315000 Ningbo, China
* Correspondence: snezana.papovic@dh.uns.ac.rs; Tel: +381-21-485-2751; Fax: +381-21-454-065

Table S1. Summarized the identification results and normalized ion currents for investigated sample of synthetized zwitterionic compound $C_1C_4imSO_3$.

m/*z* fragment	Normalized ion current for compound $C_1C_4imSO_3$
14	$1{\cdot}10^{-9}$
16	$5{\cdot}10^{-10}$
17	$2{\cdot}10^{-9}$
18	$8{\cdot}10^{-9}$
28	$1{\cdot}10^{-8}$
32	$5{\cdot}10^{-9}$
36	$4{\cdot}10^{-9}$
44	$1{\cdot}10^{-10}$
48	$2{\cdot}10^{-10}$
64	$2{\cdot}10^{-10}$

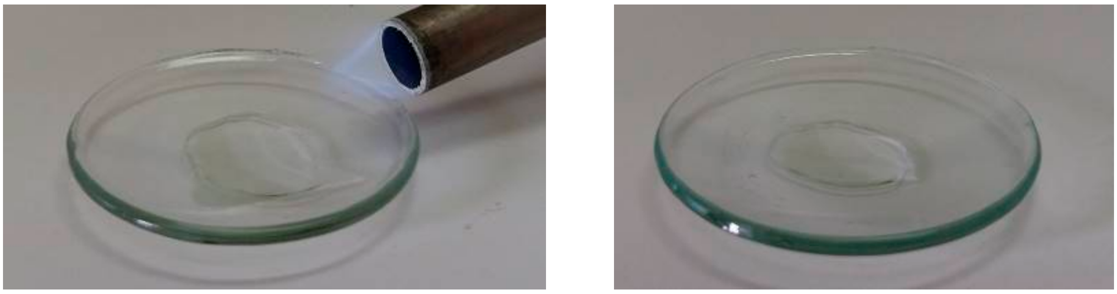

(**a**) (**b**)

Figure S1. Flammabilty test pictures of LiTFSI/C_2C_2imTFSI/$C_1C_4imSO_3$ when is: **a**) exposed to flame and **b**) after exposion of 120 s to flame.

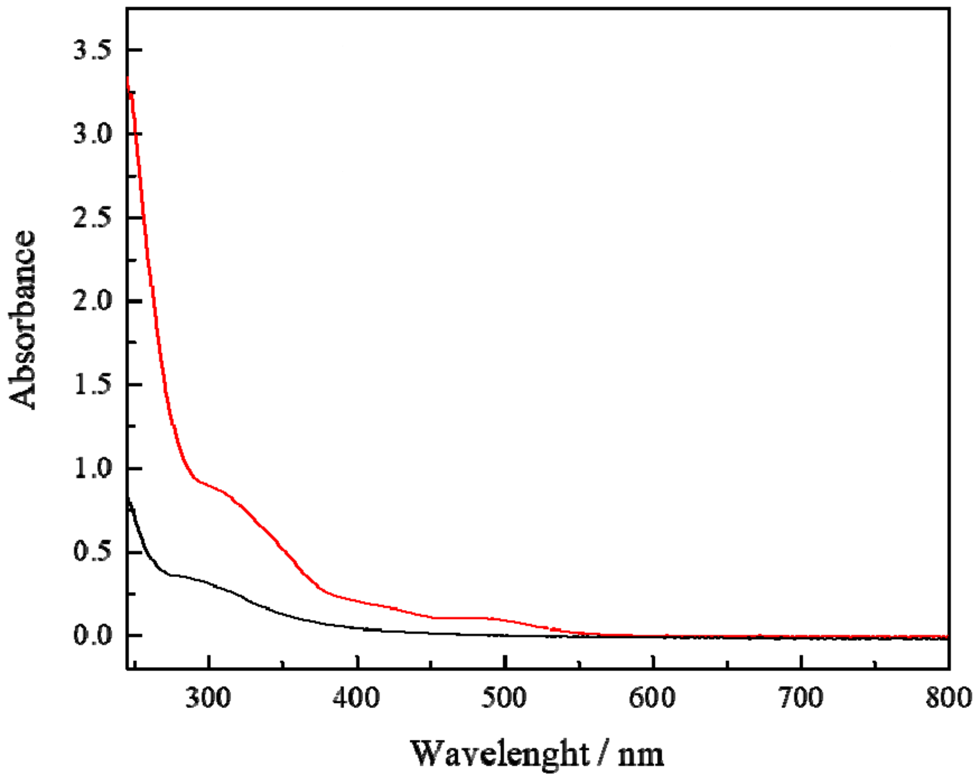

Figure S2. UV/Vis spectra of electrolyte recorded in the range from 250–800 nm at room temperature, where is red line related to the LiTFSI/C_2C_2imTFSI and the black line related to the LiTFSI/C_2C_2imTFSI/C_1C_4imSO_3 (spectra for electrolytes were recorded after galvanostatic cycling).

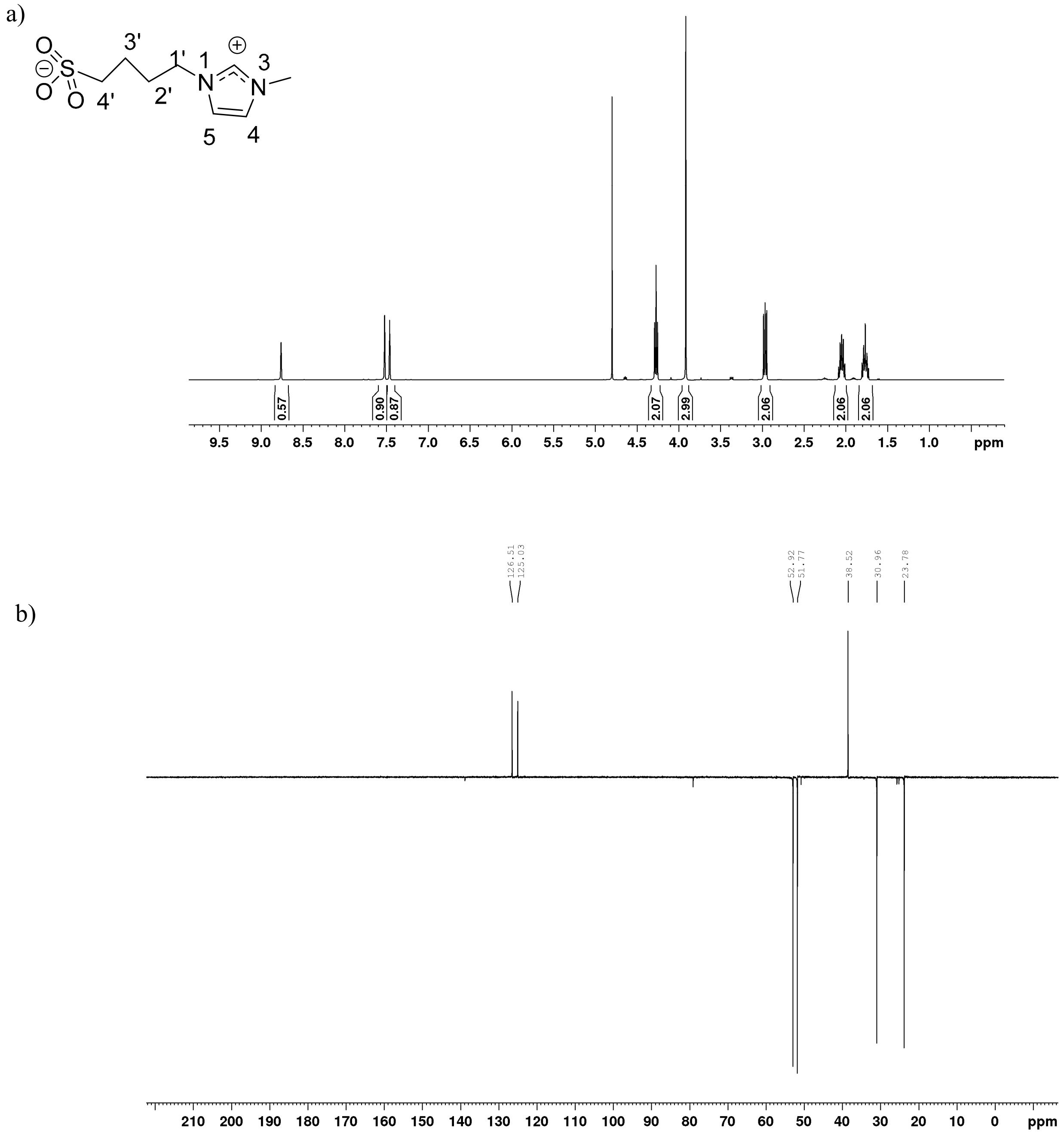

Figure S3. a) ^{1}H and b) ^{13}C NMR spectra for synthesized $C_1C_4imSO_3$

a) ^{1}H NMR: 1.76, m, 2H, $NCH_2CH_2C\mathit{H}_2CH_2SO_3^-$; 2.05, m, 2H, $NCH_2C\mathit{H}_2CH_2CH_2SO_3^-$; 2.96, m, 2H, J=7.9 Hz, $NCH_2CH_2CH_2C\mathit{H}_2SO_3^-$; 3.92, s, 3H, $NC\mathit{H}_3$; 4.27, t, 2H, J=7.1 Hz, $NC\mathit{H}_2CH_2CH_2CH_2SO_3^-$; 7.46, t, 1H, J=1.9 Hz, H-4; 7.52, t, 1H, J=1.9 Hz, H-5; 8.52, s, 0.6H, H-2.

b) ^{13}C NMR: 23.78 ($NCH_2CH_2CH_2CH_2SO_3^-$); 30.96 ($NCH_2CH_2CH_2CH_2SO_3^-$); 38.52 ($N\mathbf{C}H_3$); 51.77 ($N\mathbf{C}H_2CH_2CH_2CH_2SO_3^-$); 125.03 (C-5); 126.51 (C-4); 138.85 (C-2).

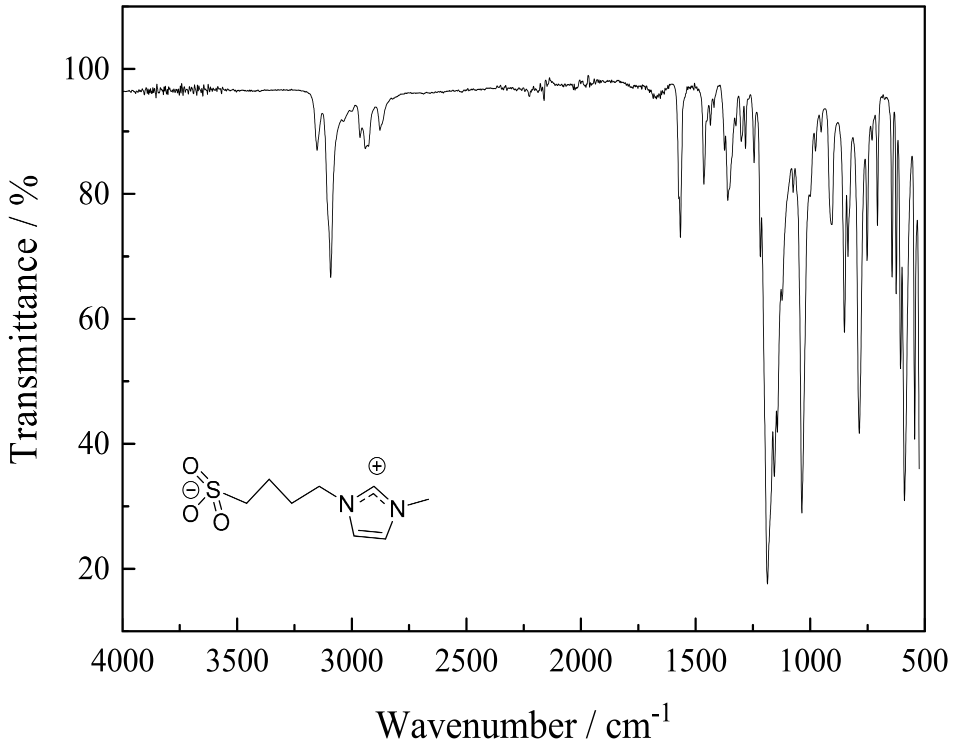

Figure S4. IR spectra of $C_1C_4imSO_3$

IR neat: 3158, 3114 (sym. stretching ν HC((4)C(5)H); 2959 (sym. stretching *ν* of imidazole ring *ν* CH_3 HCH); 2954, 2922 (asym. stretching *ν* C-H of -CH_3 and =CH_2); 2949 (asym. stretching *ν* CH_2); 1642 (stretching *ν* C=C); 1575 (in-plane *ν* of imidazolium ring); 1460 (C-N stretching of imidazole ring); 1450 (from methyl group, C-H bending); 1338 (SO_3 assymetric stretching); 1176 (sceletal *ν* of imidazolium ring); 1042 (out-of-plane C-H bending *ν* of imidazole ring); 974 (-S=O- stretching); 651 (C2-N-C5 bending *ν* of imidazole ring); 622, 607 (CCC in-plane ring bending).

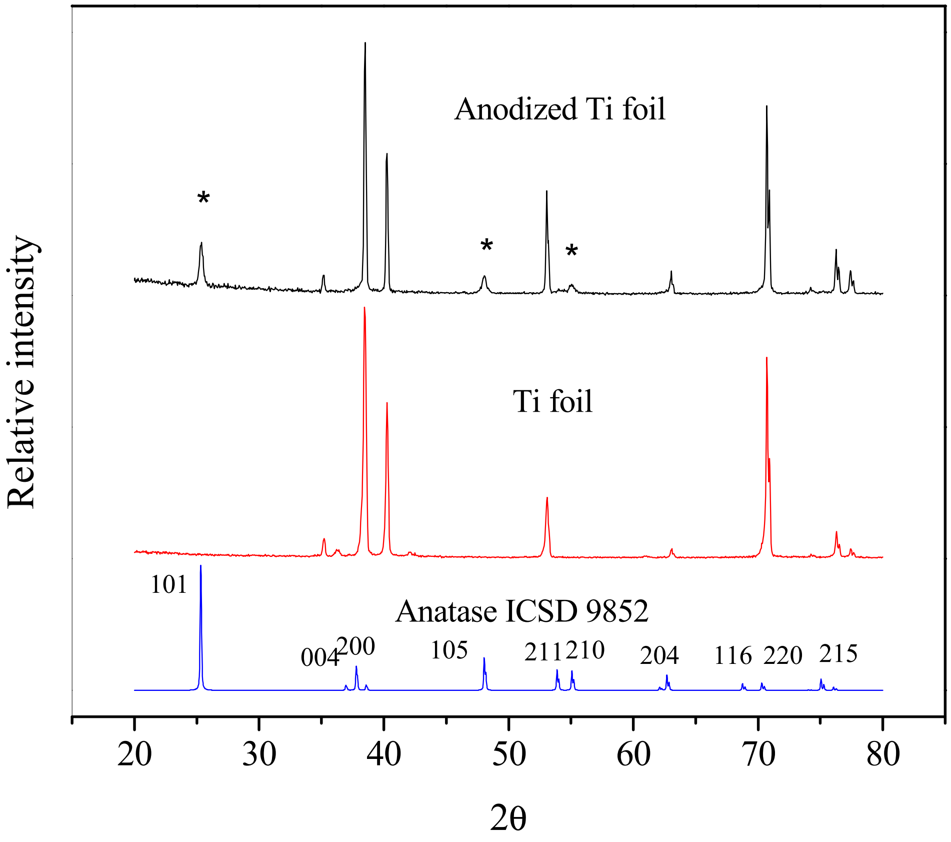

Figure S5. *X*-ray diffraction data for anodized Ti foil, Ti foil and anatase TiO_2.

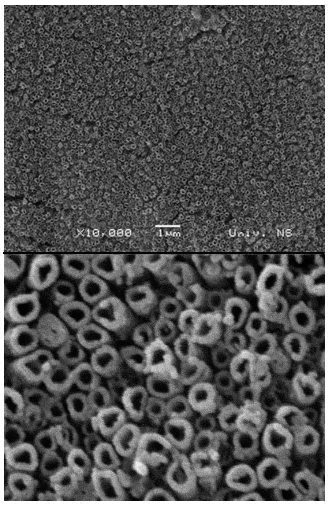

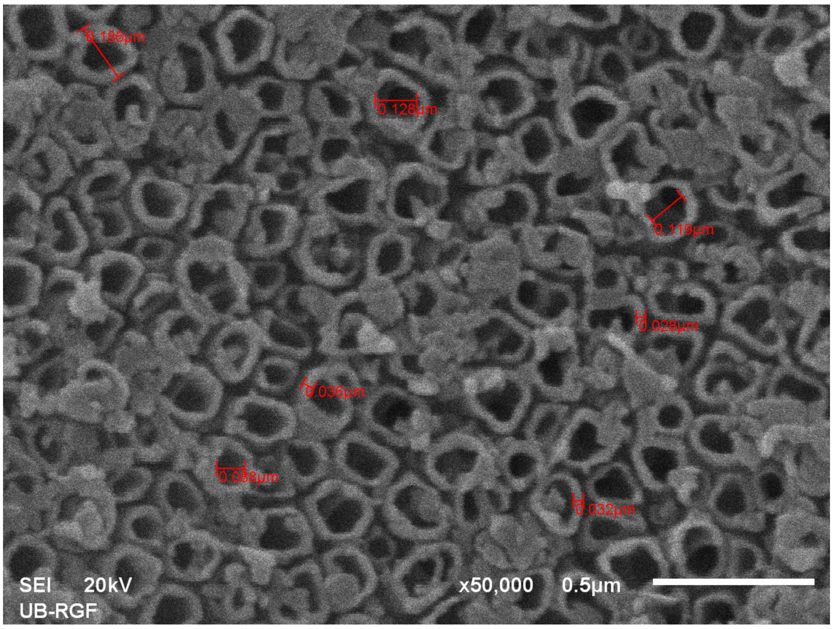

Figure S6. SEM images of TiO_2 nanotubes prepared by anodic oxidation (30 V, 6 hours) of Ti foil, with the thickness of the nanotube wall and the diameter of the nanotube opening.
